# Multifocal Tubercular Osteomyelitis: A Case with Atypical Manifestations

**DOI:** 10.1155/2011/483802

**Published:** 2011-04-27

**Authors:** Mukesh Thawani, Elizabeth Hale, Eyassu Habte-Gabr

**Affiliations:** Department of Internal Medicine, Hurley Medical Center, 2 Hurley Plaza, Suite 212, Flint, MI 48503, USA

## Abstract

Skeletal tuberculosis (TB) accounts for about 1–2% of all TB cases and 10% of extrapulmonary TB cases. We present a 19-year-old male with multifocal tubercular osteomyelitis, who presented with progressively worsening back pain, weight loss, fatigue, anorexia, decreased mobility, low-grade fever, and night sweats—but without pulmonary involvement.

## 1. Introduction

Tubercular osteomyelitis, an uncommon form of extrapulmonary tuberculosis (TB), accounts for 1% to 2% of all cases of TB and 10% of all cases of extrapulmonary TB [1].

Spinal tuberculosis accounts for 50 percent of skeletal TB cases. Thoracolumbar region is the most common site, followed by cervical and sacral vertebrae. Typically, intervertebral disc space and adjacent vertebral bodies are involved. Cases of intervertebral disc sparing have been reported [2]. However, extensive involvement of all spinal levels with extraspinal tubercular osteomyelitis is extremely rare [3, 4]. Also, very few cases of skeletal TB involve ribs [5].

Multifocal tubercular osteomyelitis rarely occurs, especially in nonimmunocompromised patients from nonendemic areas of the world with no pulmonary involvement. It must be considered in the differential diagnosis of multiple destructive skeletal lesions. This condition may mimic malignant disease clinically and radiologically. CT scans and MRI imaging can help to determine extent of bone involvement, which aids management and follow-up decisions [6]. Diagnosis is confirmed by biopsy result [7].

The extremely rare occurrence of multifocal tubercular osteomyelitis frequently causes diagnosis difficulties because low suspicion may delay appropriate treatment, resulting in devastating deformities and functional deficits [8].

## 2. Case Report

We report a 19-year-old African-American male admitted to the hospital a year into his incarceration in prison. He described 6 months' history of progressively worsening back pain, weight loss, fatigue, anorexia, decreased mobility (making him wheelchair-bound), and associated low-grade fever with night sweats. He denied history of cough or hemoptysis, though family history included pulmonary tuberculosis. Prison TB skin test (PPD) was negative. He denied history of smoking, alcohol consumption or drug abuse.

Upon physical exam, the patient appeared malnourished, tachycardic, and had a low-grade fever. He was reluctant to move due to severe pain, with extreme cervical tenderness and tender swelling at the lumbosacral region. Chest was clear with normal breath sounds; cardiovascular exam was unremarkable. Abdomen was soft with no organomegaly.

**Figure 1:**
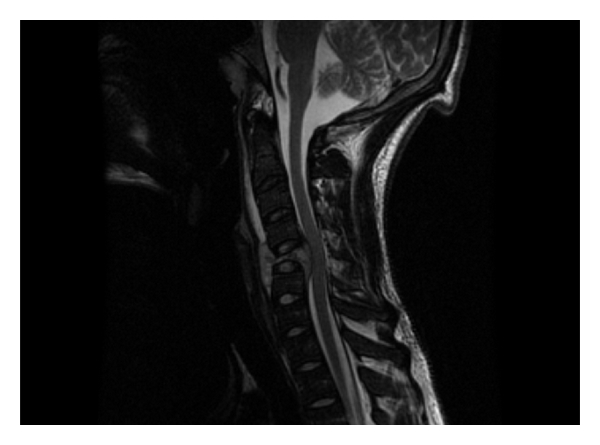
Sagittal T2W image shows collapse of C5 with preservation of adjacent disc spaces. Soft tissue mass extends posteriorly into the anterior epidural spaces, compressing on the spinal cord.

**Figure 2:**
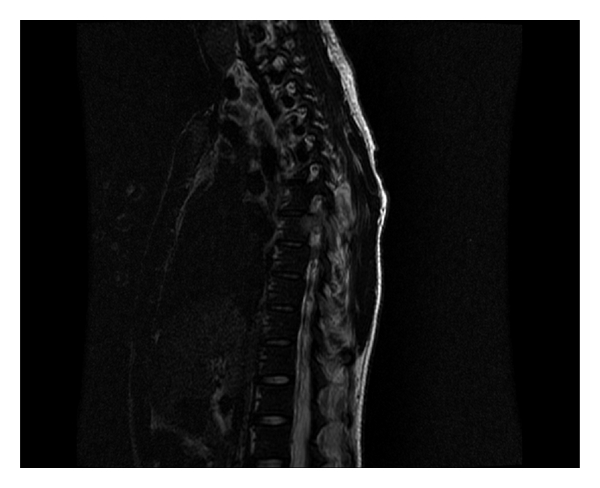
Sagital T2W image shows abnormal signal within the right pedicle of T6 with associated soft tissue. Similar abnormality was noted in the left pedicle of T8.

**Figure 3:**
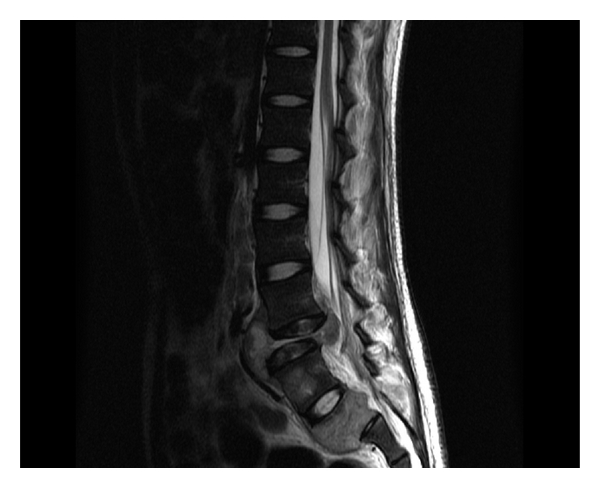
Sagital T2W image shows collapse and destruction of L5 vertebral body with associated soft tissue extending anteriorly into prevertebral region and posteriorly into anterior epidural space. There is also similar soft tissue involving the S2 vertebral body (there is lumbarisation of S1).

Other significant findings included straight leg-raising was positive on right. Review of CNS was normal with no neck stiffness. Sensory system and reflexes were normal but motor system had reduced power of right lower extremity. Lab findings were significant for high erythrocyte sedimentation rate (120), high C-reactive protein (12), negative blood cultures, negative PPD, and negative HIV antibody tests. Chest X-ray was normal. MRI of spine showed destructive lesions involving the body of C5, T6 (right pedicle), T8 (left pedicle) and adjoining posterior ribs, L5, S1, S2, and left iliac bone (Figures 1, 2, and 3). Intervertebral discs spared. Bone scan revealed multiple areas of increased radiotracer activity in midthoracic spine and adjacent ribs, lumbar spine, and left ileum. 

At this time, differential diagnoses included lymphoma, eosinophilic granuloma, myeloma, and metastatic disease. Then, serum protein electrophoresis revealed polyclonal gammopathy; Bence Jones proteins were negative. A CT-guided biopsy of the left iliac bone lesion showed granulomatous inflammation with giant cells and rare acid-fast bacilli. Final culture report was positive for mycobacterium tuberculosis. 

Patient was diagnosed with multifocal tubercular osteomyelitis and was started on quadruple antitubercular treatment, including rifampicin, isoniazide, ethambutol, and pyrazinamide. Patient then underwent surgical decompression of cervical spine. Finally, he was transferred to University of Michigan for surgical decompression of lumbosacral spine. He was transferred back to prison, where he completed the course of anti-TB treatment. According to prison officials two years after the first presentation, he was doing better and was mobilizing without any support. 

## 3. Discussion

Immunocompromised patients have an increased risk in developing extrapulmonary tuberculosis. Prevalence is higher, especially in HIV-infected, hemodialysis patients and patients who are on immunosuppressive therapy [1]. Multifocal skeletal tuberculosis is less common in nonimmunocompromised patients. This case report describes an unusual case of multifocal tubercular osteomyelitis without an underlying disorder. The insidious nature of this form of skeletal TB often leads to delayed or missed diagnosis, with devastating consequences for the patient [8].

In a typical case of tubercular osteomyelitis, two or more contiguous vertebrae are involved because one intervertebral artery supplies two adjacent vertebrae, allowing hematogenous spread [3]. The published medical literature shows only a few reported cases with extensive involvement of multiple spinal regions. Our patient presented with tuberculous involvement at multiple spinal levels, including cervical, thoracic, lumbar, and sacral vertebrae, which is extremely rare. 

Two distinct patterns of spinal TB can be identified from CT and MRI findings: the first (typical) pattern involves intervertebral disc and adjacent vertebral bodies, the second (atypical) pattern is characterized by involvement of the body or neural arch of one or more vertebrae with sparing of intervertebral disc [2]. Our case report shows the extremely rare case of multifocal spinal and extraspinal involvement, with sparing of disc space in a nonimmunocompromised patient without pulmonary involvement, which mimics primary or secondary malignant process. Therefore, this case emphasizes the importance of keeping the diagnosis of TB in mind as it can present in multiple unusual sites.

Tuberculous osteomyelitis of the rib is rare. The spine is the most common site and accounts for more than 40–60% of all cases of skeletal TB. Usual locations are lumbar and thoracic, with cervical involvement only 2–3% of the time. The ribs are involved in only 0.1% of all tuberculosis infections [5]. Our case also had rare involvement of posterior thoracic rib. In most patients, this finding, along with multiple osteolytic lesions, can be mistaken for malignancy, as occurred in our case. A high degree of suspicion is the key in diagnosing patients presenting with vague systemic features and multiple destructive bone lesions.

## 4. Conclusion

 Multifocal tubercular osteomyelitis must be considered in the differential diagnosis of patients who present with multiple destructive osseous lesions, which may be associated with intervertebral disc sparing. Clinical and radiological findings may be indistinguishable from malignant disease. A high index of suspicion may prevent delayed diagnosis, and early treatment may prevent subsequent complications. A negative tuberculin skin test does not rule out the possibility of tuberculous bone involvement. Also a lack of radiographic and clinical evidence of pulmonary involvement does not rule out skeletal TB. Finally, a definitive diagnosis requires the isolation of *M. tuberculosis*.

##  Conflict of Interests

The authors report no conflict of interests with people or organizations that could influence or bias this paper.
